# Isolated exopolysaccharides from *Lactobacillus rhamnosus* GG alleviated adipogenesis mediated by TLR2 in mice

**DOI:** 10.1038/srep36083

**Published:** 2016-10-27

**Authors:** Zhen Zhang, Zhigang Zhou, Yu Li, Linkang Zhou, Qianwen Ding, Li Xu

**Affiliations:** 1 Key Laboratory for Feed Biotechnology of the Ministry of Agriculture, Feed Research Institute, Chinese Academy of Agricultural Sciences, Beijing, P. R. China; 2 MOE Key Laboratory of Bioinformatics and Tsinghua-Peking Center for Life Sciences, School of Life Sciences, Tsinghua University, Beijing 100084, P. R. China

## Abstract

The fibroblast cell line of 3T3-L1 was used as a cell model for screening and evaluating the feasibility of probiotic components in improving animal lipid metabolisms. The extracts from 12 *Lactobacillus* strains caused significantly reduced triacylglycerol (TAG) accumulation but with severe inflammation induction in 3T3-L1 adipocytes. Interestingly, exopolysaccharides (EPS) from LGG (*Lactobacillus rhamnosus* GG) significantly decreased the TAG accumulation without any inflammation. The anti-obesity effect of EPS was confirmed in high-fat-diets feeding mice. Fat pads of mice injected with EPS (50 mg/kg) every two days for two weeks were significantly reduced with much smaller adipocytes, compared with the counterparts. The levels of TAG and cholesterol ester in liver, as well as serum TAG, were decreased in EPS injected mice. In addition, down-regulated inflammation was observed in adipose tissue and liver. Interestingly, the expression of TLR2 in adipose tissue and 3T3-L1 cells was significantly increased by EPS addition. Moreover, the reverse of TAG accumulation in TLR2 knockdown 3T3-L1 in the presence of EPS confirmed that the inhibition effect of EPS on adipogenesis was mediated by TLR2. EPS from LGG has the potential for therapeutic development to intervene lipid metabolic disorders in mammals.

As a worldwide epidemic associated with many metabolic diseases, obesity imposes an enormous burden on individual and public health^1^. The obesity development is a complex process involving genetic, environmental, neural and endocrine factors^2^, and even infectious agents^3^. Moreover, recent studies have shown that obesity, as a transmissible phenotype by microbiota transplantation, is associated with specific structural and functional configurations of the bacterial gut microbiota^4^,^5^. The increase of some bacterial groups, mainly *Bifidobacterium* genus, has been associated with lean status^6^,^7^,^8^,^9^, while other bacterial groups, such as *Staphylococcus aureus*^9^,^10^ and *Escherichia coli*^10^,^11^, have been associated with obesity.

Probiotics are live microorganisms that, when administered in proper amounts, improve host health^12^. LGG, isolated from the healthy adult’s faeces^13^,^14^, has been intensively studied and showed several beneficial effects. Most studies about LGG focused on its effects on modulating inflammatory responses and its anti-inflammatory mechanisms *in vitro*^15^,^16^ or *in vivo*^17^,^18^,^19^. Recently, LGG treatment was reported to improve insulin sensitivity and reduce body weight in HFD-fed mice through increased adiponectin production^20^. Ji *et al*. found that LGG treatment did not affect body weight of mice, but resulted in a significant reduction in epididymal fat^21^. However, Park *et al*.^22^ reported that LGG could protect mice against adiposity only when treated after dysbiosis induced by HFD feeding, but this was not the case when LGG was co-treated from the beginning of HFD feeding. All these studies above were performed by orally administering intact LGG cells to HFD-fed mice daily. In spite of these apparent anti-obesity effects of LGG, questions still remain in elucidation of the mechanism underlying the effects. In addition, nowadays the mechanisms of the anti-obesity effects of *lactobacillus* mainly focus on changing gut microbiota^21^,^23^ and *lactobacillus*-derived metabolites, such as SCFA^24^,^25^, and conjugated linoleic acid^26^. However, the roles of the components or molecules from lactobacillus itself are ignored.

Cell wall polysaccharides are ubiquitous components of the cell envelope of lactobacilli. These polysaccharides, which are generally neutral, are found covalently attached to peptidoglycan glycan strands, loosely associated with the cell envelope or released into the extracellular medium as EPS^27^. *Lactobacillus*-produced EPSs displayed encouraging therapeutic potential in terms of immunomodulation, hypocholesterolemia, antioxidant properties, anti-tumor activity and anti-atherosclerotic effects^28^,^29^. Polysaccharides of lactobacilli are generally heteropolysaccharides of complex structures differing in the nature of sugar monomers, the modes of linkage, branching and substitutions^27^. Since *Lactobacillus*-produced EPSs are so structurally diverse, it is perhaps not surprising that a wide variety of biological activities described above and more beneficial effects are waiting to be discovered. Therefore, we aimed to systematically evaluate whether EPS from LGG was able to functionally regulate host’s lipid metabolism and further explored the possible mechanisms in adipocytes and mouse models.

## Results

### Cell extracts from LGG inhibited the TAG accumulation but induced inflammation in 3T3-L1 cells

The cell extracts from 12 candidate *Lactobacillus* strains were used to detect the effects on adipocyte adipogenesis in 3T3-L1 cell model. The 3T3-L1 cells were treated with 40 μg/ml cell extracts from these *Lactobacillus* strains at the initiation of preadipocyte differentiation (day 0)^30^,^31^. The intracellular TAG storage in 3T3-L1 was significantly inhibited by the extracts from all the 12 strains (Supplementary Fig. S1A).

Then, the inflammation status were examined in 3T3-L1 cells in order to explore whether these *Lactobacillus’* extract decreased intracellular TAG content by affecting the normal physiological state of 3T3-L1. The expression of M1 inflammatory genes, including TNF-a, MCP-1 and IL-6 as proinflammatory markers, was significantly increased by LAB extract in mature 3T3-L1 adipocytes (Supplementary Fig. S1B). In contrast, the expression of the M2 anti-inflammatory gene markers, such as arginase 1, MGL1, and Clec7a, was reduced in response to the treatments (Supplementary Fig. S1C). These results indicated that it was necessary to detect both adipogenesis and inflammation for the proper evaluation of probiotic effects in adipocytes.

**Figure 1:**
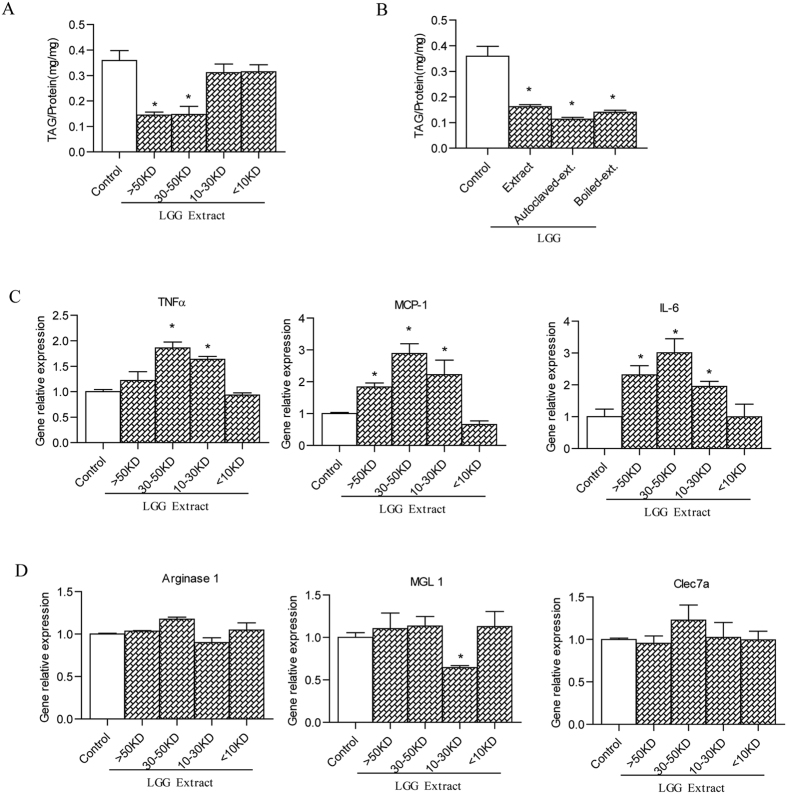
The effects of cell extract from LGG on the adipogenesis (**A,B**) and inflammation (**C,D**) in 3T3-L1 adipocytes. 3T3-L1 cells from initiating differentiation (Day 0) to terminate mature (Day 6) as indicated in methods were treated with the supernatant of cell extracts (40 μg/ml) from LGG or water as the control. (**A**) Effects of four fractions of LGG extract (<10 KD, 10–30 KD, 30–50 KD and >50 KD) on TAG accumulation in 3T3-L1 adipocyte. (**B**) Effects of LGG extracts after boiling or autoclaving on TAG accumulation in 3T3-L1 adipocyte. The expression of M1 proinflammatory genes (**C**) and M2 anti-inflammatory genes (**D**) in 3T3-LI cells by q-PCR. Data were expressed as mean ± SEM of three independent experiments (n = 3). Significance was established using a two-tailed Student’s t-test. Differences were considered significant at *P* < 0.05 (*) and *P* < 0.01 (**).

Among the 12 strains, LGG extracts induced the lowest inflammation induction (Supplementary Fig. S1B,C). Therefore, LGG was chosen for subsequent investigation. Four fractions of LGG extract with different molecular weight ranges (<10 KD, 10–30 KD, 30–50 KD and >50 KD) were obtained by ultrafiltration device. The fractions of 30–50 KD and >50 KD significantly decreased the intracellular TAG contents of 3T3-L1 cells (Fig. 1A), with no effect observed in the fractions <10 KD and 10–30 KD. It indicated that the macromolecules from LGG fractions reduced the TAG accumulation. Moreover, as shown in Fig. 1C,D, expression of M1 inflammatory genes, such as TNF-a, MCP-1, and IL-6 was significantly increased by the fractions of 30–50 KD of LGG extract in mature 3T3-L1 adipocytes, and M2 anti-inflammatory genes, including Arginase 1, MGL1, and Clec7a, were not significantly changed by LGG extract administration in 3T3-L1 culture. In addition, the boiled or autoclaved LGG extracts after boiling both triggered the inhibited effects on TAG accumulation similar to untreated extracts (Fig. 1B). Therefore, we propose that some stable macromoleculars from LGG extracts, such as EPS, may play an important role in reducing the intracellular TAG accumulation of 3T3-L1.

### Isolation, purification and characterization of the EPS from LGG

**Figure 2:**
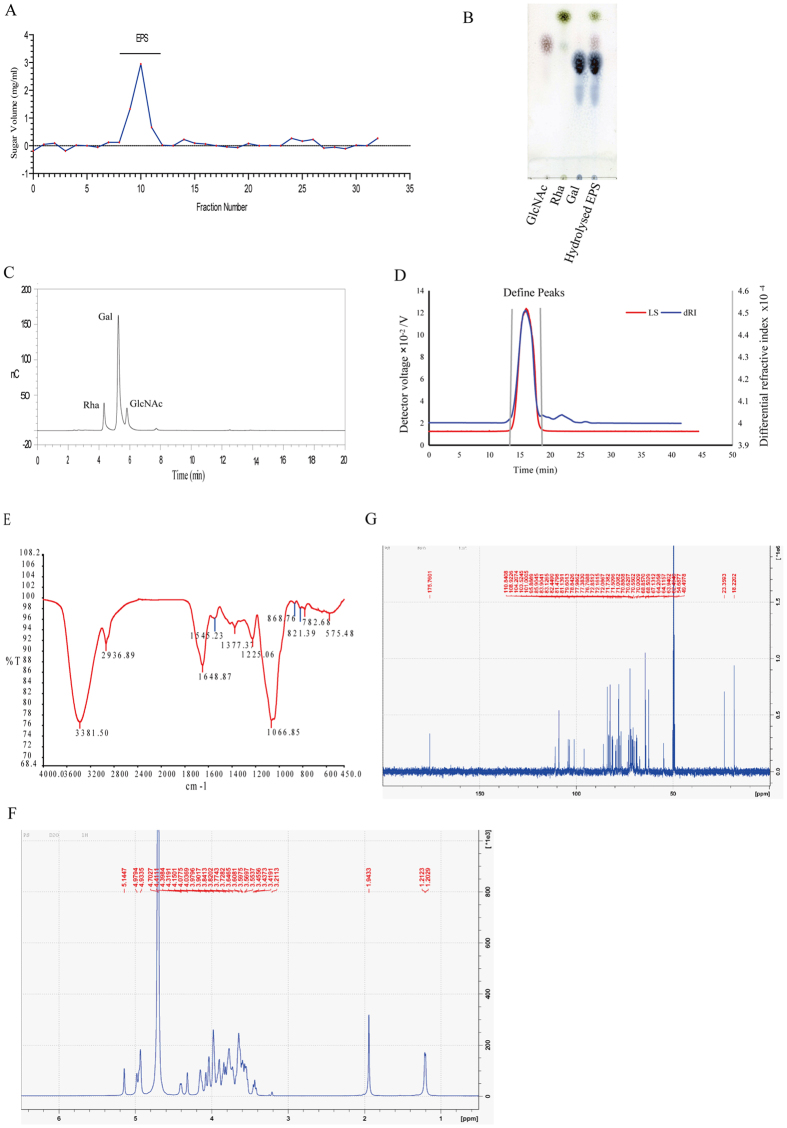
Isolation, purification and structural characterization of the exopolysaccharide from LGG. (**A**) Purification of LGG EPS by size-exclusion chromatography on a column of Superdex75 (10/300 GE). (**B**) TLC profile of monosaccharide composition of LGG EPS (GlcNAc, N-acetyl-D-glucosamine; Rha, rhamnose; Gal, galactose). (**C**) HPAEC profile of monosaccharide composition of LGG EPS. (**D**) Gel permeation chromatography-MALLS of LGG EPS. (**E**) IR spectrum of LGG EPS. NMR spectrum of LGG EPS (**F**) ^1^H-NMR and (**G**) ^13^C-NMR.

Thereafter, LGG EPS was isolated, purified and characterized. The crude EPS was further purified by size-exclusion chromatography on a column of Superdex75 (10/300 GE). As shown in Fig. 2A, purified LGG EPS appeared as a single and symmetrical peak, indicating that EPS was a homogeneous polysaccharide.

Monosaccharide composition of EPS was analyzed by TLC and HPAEC. According to TLC, EPS was composed of rhamnose, N-acetyl-D-glucosamine and galactose (Fig. 2B). The hydrolyzate of EPS in HPAEC tended to have three peaks (Fig. 2C).They were identified as rhamnose, N-acetyl-D-glucosamine and galactose by comparing with their standards, consistent with the result of TLC. Therefore, it was confirmed that EPS was the polymers of rhamnose, N-acetyl-D-glucosamine and galactose.

The molecular weight of the purified EPS determined by GPC/MALLS was 229,300 ± 0.008 kDa. The GPC/MALLS profile (Fig. 2D) demonstrated a single and symmetrically sharp peak of EPS, revealing that it was a homogeneous polysaccharide. The polydispersity Mw/Mn 1.268 (Supplementary Table S2) (~1.00) also indicated that EPS was a narrow distribution sample.

The FT-IR spectra of EPS exhibited a variety of typical absorption peaks of polysaccharides, as shown in Fig. 2E. Bands were visible at approximately 3400 cm^−1^, 2939 cm^−1^ and 990–1200 cm^−1^, which are common to all polysaccharides and represent O-H stretching, C-H stretching of the -CH2 groups and polysaccharides, respectively^32^.

The ^1^H NMR spectroscopy of EPS (Fig. 2F) was complex due to the convergence of most sets of the signals in the region 3.21–4.41 ppm. The signal at δ 1.94 ppm indicated that EPS was acetylated^33^. The ^13^C NMR spectrum of EPS is shown in Fig. 2G. In the anomeric region, six major carbon resonances appeared between 90.00 ppm and 110.00 ppm. Thus, the repeating unit of the EPS contains six sugar residues. According to the literature, signals at δ 23 ppm and 175 ppm nearby arose from carbon of the methyl and carbonyl groups on acetyl group, respectively^34^. Based on the results of monosaccharide compositions, FT-IR spectroscopy and NMR analyses, We can confirm the EPS produced by LGG in our study is fully consistent with previously published structural studies by Landersjö *et al*.^35^. Furthermore, we enriched the information on the molecular weight and molecular weight distribution of the EPS (Fig. 2D).

### Inhibited adipogenesis in 3T3-L1 cells by LGG EPS without pro-inflammatory induction

**Figure 3:**
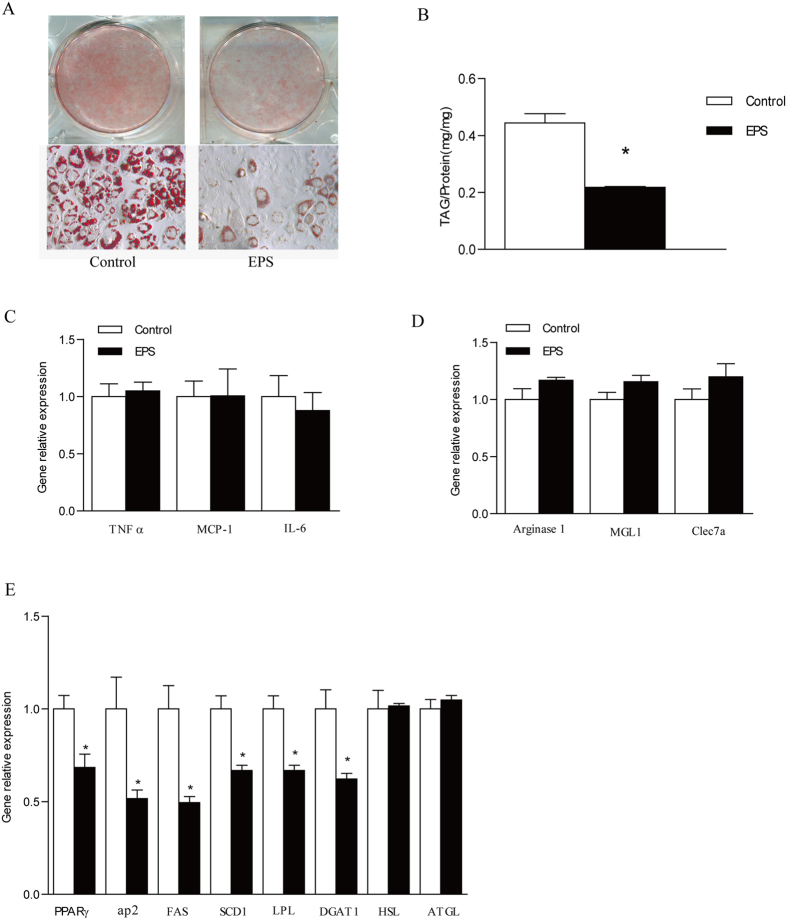
Inhibited adipogenesis in 3T3-L1 cells by LGG EPS without proinflammation induction. The cell line of 3T3-L1 was treated by isolated EPS from LGG during the Day 0–6 and the sampling was done on Day 6 for TAG and protein assay or total RNA extraction. (**A**) The profile of lipid droplet formation with Oil red O staining in 3T3-L1 adipocytes. (**B**) TAG accumulation in 3T3-L1 adipocyte by TAG assay. (**C–E**) The expression of M1 proinflammatory genes (**C**) M2 anti-inflammatory genes (**D**) and lipid metabolism related genes in 3T3-LI cells by q-PCR (**E**). Data were expressed as mean ± SEM of three independent experiments (n = 3). Significance was established using a two-tailed Student’s t-test. Differences were considered significant at *P* < 0.05 (*) and *P* < 0.01 (**).

To evaluate the effects of LGG EPS on the adipogenesis in adipocytes, the 3T3-L1 cells were treated with 10.0 μg/ml EPS during the whole differentiation periods (Day 0–6). Significant reduction of lipid accumulation was visualized with Oil Red O staining compared to the control (Fig. 3A). The result of TAG assay also revealed that EPS caused a 50% reduction of intracellular TAG levels in 3T3-L1 cells (Fig. 3B). The preadipocytes from subcutaneous (SAT) and visceral (gonadal,) fat depots of C57BL/6J male mice fed with normal diets (ND) were isolated and cultured with EPS administration. Similar to the result in 3T3-L1, EPS also inhibited adipogenesis in primary adipocytes (Supplementary Fig. S2).

Consistently, EPS significantly decreased the gene expression of adipogenesis markers in 3T3-L1 cells, including PPARγ, ap2, FAS, SCD1, LPL, DGAT1, but had no effects on the expression of genes involved in lipolysis (HSL, ATGL) (Fig. 3E). It indicated that that EPS reduce TAG accumulation by inhibiting lipogenesis. Moreover, as shown in Fig. 3C,D, expression of M1 inflammatory genes, such as TNF-α, MCP-1, and IL-6, and M2 anti-inflammatory genes, including Arginase 1, MGL1, and Clec7a, were not significantly changed by EPS administration in 3T3-L1 culture. Therefore, isolated LGG EPS reduce the adipogenesis of 3T3-L1 adipocytes with no induction of inflammation.

### LGG EPS, not its degraded monosaccharide, inhibited the adipocyte differentiation

**Figure 4:**
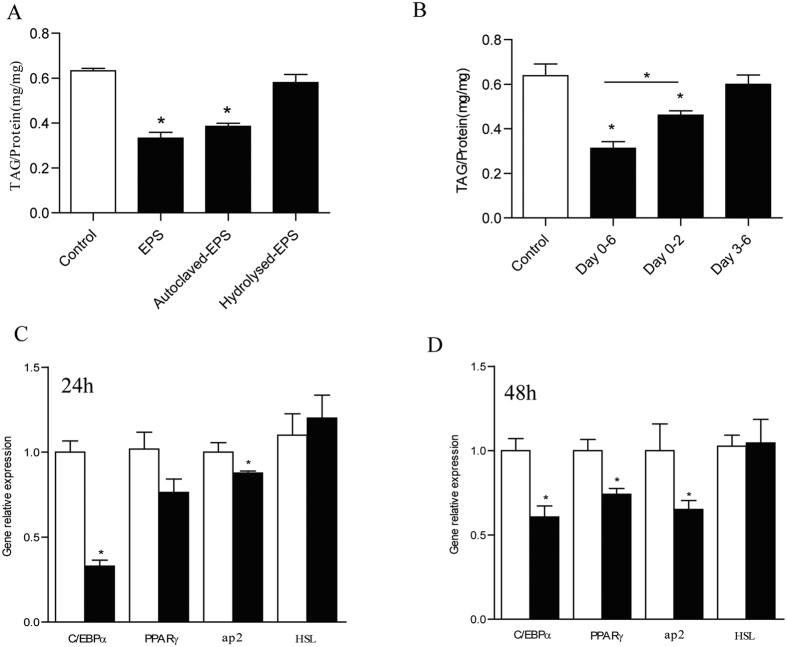
LGG EPS, not degraded EPS, inhibited the preadipocyte differentiation. (**A**) TAG accumulation in 3T3-L1 adipocyte treated by LGG EPS, autoclaved EPS or its degraded products by sulfuric acid. These different forms of EPS or its degraded products were administrated to 3T3-L1 cells during Day 0–6 and the sampling was done on Day 6 for TAG and protein assay. (**B**) TAG accumulation in 3T3-L1 adipocytes treated by LGG EPS at different differentiation periods as indicated. 3T3-L1 cells were treated by LGG EPS during Day 0–2, Day 3–6 and Day 0–6 and the sampling was done on Day 6 for TAG and protein assay. (**C,D**) The expression of lipid metabolism related genes when 3T3-L1 cells were treated by LGG EPS during Day 0–1 (namely 24 hours, **C**) and Day 0–2 (namely 48 hours, **D**). The sampling was done immediately after the treatments finished for RNA extraction and consequent qPCR. Data were expressed as mean ± SEM of three independent experiments (n = 3). Significance was established using a two-tailed Student’s t-test. Differences were considered significant at *P* < 0.05 (*) and *P* < 0.01 (**).

To analyze the functional effects of EPS from LGG, the isolated EPS, autoclaved EPS or the products of degraded EPS (by sulfuric acid) were used to treat 3T3-L1 from preadipocytes (2-day confluence, indicated as day 0) to mature adipocytes (day 6). As shown in Fig. 4A, the autoclaved EPS also significantly decreased the intracellular TAG storage, similar to its unautoclaved form. However, hydrolyzed EPS products, mainly composed of galactose, rhamnose and N-acetyl-D-glucosamine, had no significant effects on 3T3-L1 adipogenesis. The data indicated that the EPS itself, rather than the monosaccharides hydrolyzed from LGG EPS, played the role in inhibiting the intracellular TAG storage.

The differentiation of 3T3-L1 cells from preadipocytes to mature adipocytes is divided into two stages, including cell monoclonal expansion to initiate the differentiation and terminal maturation. In order to study which stage was important for EPS to inhibit adipogenesis, EPS was administrated respectively during 3T3-L1 initiating differentiation stage (Day 0–2), maturation stage (Day 3–6) or the whole stage of differentiation (Day 0–6). The significantly deceased adipogenesis could be observed by 2-day treatment of EPS in initiating differentiation stage, but not occurred in the 4-day treatment of terminal maturation stage, indicating the dominant roles of EPS in initiating differentiation stage. Cell counting assay demonstrated that EPS administration did not change the cell numbers in initiating differentiation stage which was regarded as adipocyte monoclonal expansion period (Supplementary Fig. S3). The inhibition of TAG accumulation were further promoted when EPS was added during the whole differentiation stage, compared with EPS treatment only at the initiating differentiation stage. This suggested that that durable administration in terminal maturation following initiating differentiation was necessary to further decrease the TAG accumulation (Fig. 4B). C/EBP family and PPARγ control the adipocyte differentiation as the key transcriptional regulators, which are responsible for different differentiation stages. To monitor the effective process of EPS, the cells were sampled for gene expression analysis immediately after EPS treatments were finished. The 24-hour EPS treatment during initiating differentiation stage significantly decreased C/EBPα expression (Fig. 4C), which is known as early differentiation regulator of the adipocytes. The significantly decreased expression of PPARγ was observed till 48-hour EPS treatment in initiating differentiation stage (Fig. 4D). The downstream gene expression, ap2, was also significantly decreased. These gene expression profiles indicated that the EPS treatment was sensed in the initiating differentiation stage, consistent with decreased TAG by EPS administration in this period.

### LGG EPS suppressed the increase in body fat in HFD-feeding C57BL/6J mice

**Figure 5:**
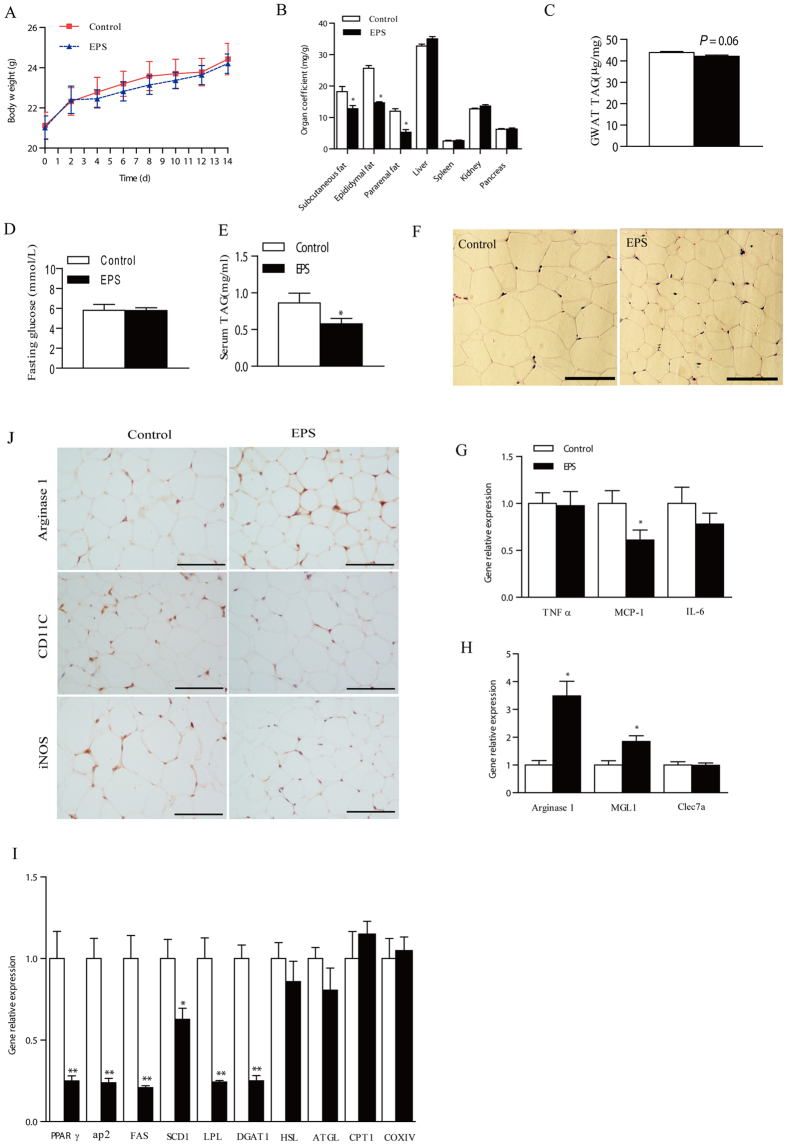
LGG EPS administration improved mouse lipid metabolism in adipose tissue and serum. 6-week-old C57BL/6J mice with HFD-feeding were intraperitoneally injected with 50 mg/kg of EPS or saline as control every two days (n = 6). 14 days later, the mice were sacrificed for sampling. (**A**) Body weight profile during the 14 days (n = 6). (**B**) Weight ratio of indicated tissue or organ to body (n = 6). (**C**) The relative TAG levels in GWAT (n = 6). (**D**) Fasting glucose level (n = 6). (**E**) Serum TAG level (n = 6). (**F**) Represented image of GWAT histology with H&E staining (n = 3). The scale bar is 100 μm. The expression of M1 proinflammatory genes (**G**) and M2 anti-inflammatory genes (**H**) and lipid metabolism related genes (**I**) in GWAT measured by q-PCR (n = 6). (**J**) Arginase 1, CD11C, and iNOS levels by immunohistochemical analysis in the GWAT of C57BL/6J mice (n = 3). The scale bar is 100 μm. Significance was established using a two-tailed Student’s t-test. Differences were considered significant at *P* < 0.05 (*) and *P* < 0.01 (**).

To confirm the effects of the EPS *in vivo*, 50 mg/kg of EPS or saline as control was intraperitoneally injected to HFD-feeding C57BL/6J mice every two days for two weeks. No significant changes were observed in weight gain (Fig. 5A), food and water intake following EPS injection (Supplementary Fig. S4). However, EPS injection significantly decreased fat mass, including subcutaneous (0.29 ± 0.06 v.s. 0.42 ± 0.09 g, P < 0.05), gonadal (0.33 ± 0.02 v.s. 0.58 ± 0.07 g, P < 0.05) and pararenal (0.13 ± 0.05 v.s. 0.28 ± 0.06 g, P < 0.05) white adipose tissue, compared with the control. No weight changes were observed in other abdominal organs including liver, spleen, kidney, pancreas (Fig. 5B). The HE staining showed that the adipocyte size in GWAT was much smaller in the treatment group than control (Fig. 5F). The relative TAG contents in GWAT were not changed (Fig. 5C). Fasting glucose concentrations were also similar in both groups (Fig. 5D). However, the serum TAG levels were significantly reduced by EPS treatment (Fig. 5E). Consistently, the expression of genes involved in adipose differentiation and adipogenesis were significantly inhibited, without significant changes in lipolysis (Fig. 5I). Moreover, the expression of M1 inflammatory genes, such as MCP-1 and IL-6, was significant inhibited in GWAT by EPS administration (Fig. 5G). On the contrary, the expression of M2 anti-inflammatory genes, arginase 1 and MGL1, was increased by EPS treatment (Fig. 5H). Immunohistochemical analyses also confirmed that CD11C and iNOS (markers of M1-like macrophages) protein levels were significantly lower in the GWAT of EPS-treated mice, while protein levels of arginase 1 (a marker of M2-like macrophages) were significantly increased (Fig. 5J). Together, these results indicated that EPS injection reduced inflammation. In addition, No effects of EPS injection were observed in the mice fed with normal diets (Supplementary Fig. S5).

### EPS reduced the lipid accumulation in the liver

**Figure 6:**
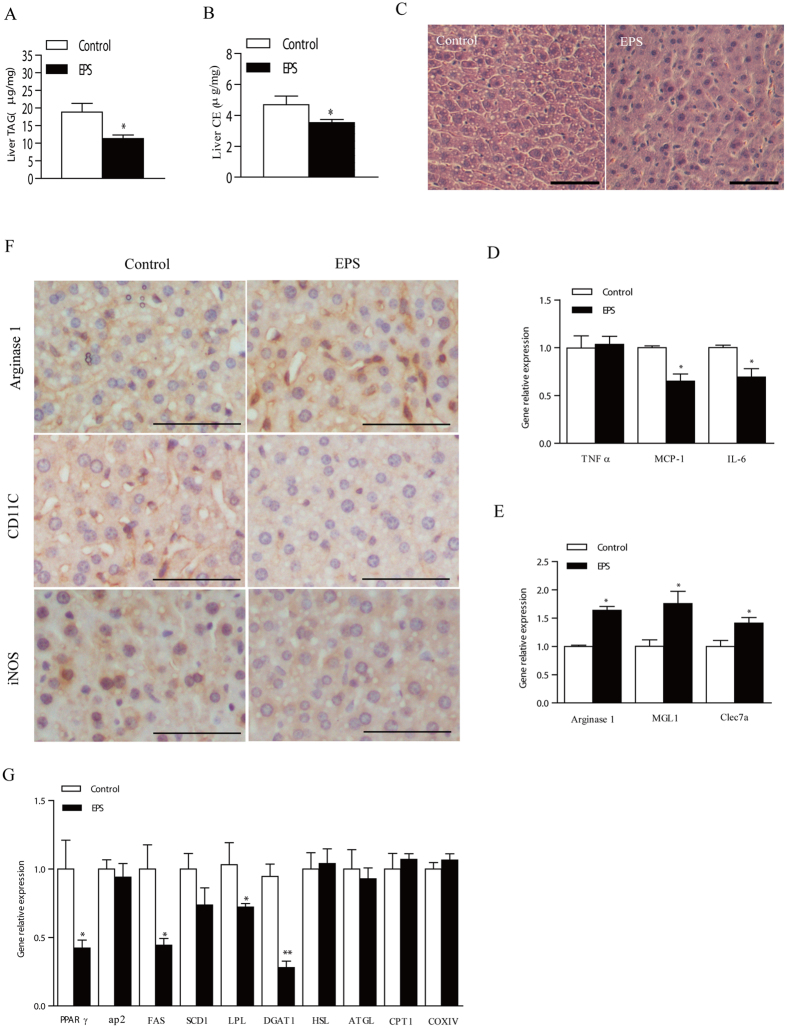
LGG EPS administration improved mouse lipid metabolism in liver. 6-week-old C57BL/6J mice with HFD-feeding were intraperitoneally injected with 50 mg/kg of EPS or saline as control every two days (n = 6). 14 days later, the mice were sacrificed for sampling. (**A,B**) Relative contents of TAG (**A**) and cholesterol ester (**B**) in liver (n = 6). (**C**) Representative image of HE liver histology with H&E staining (n = 3, scale bar = 50 μm). (**D–F**) The expression of M1 proinflammatory genes (**D**) M2 anti-inflammatory genes (**E**) and lipid metabolism related genes (**G**) in livers measured by q-PCR. (F) Arginase 1, CD11C, and iNOS levels by immunohistochemical analysis in liver of C57BL/6J mice (n = 3). The scale bar is 50 μm. Data are expressed as mean ± SEM of three independent experiments (n = 3). Significance was established using a two-tailed Student’s t-test. Differences were considered significant at *P* < 0.05 (*) and *P* < 0.01 (**).

The lipid metabolism in liver was determined after EPS injection. The contents of TAG and cholesterol ester (CE) in livers from the EPS group were significantly reduced compared with control group infected with saline (Fig. 6A,B). The contents of TAG in muscles, intestines, pancreases, spleens, kidneys and hearts from the EPS group were not significantly increased (Supplementary Fig. S6). Consistently, the number of lipid droplets in EPS-treated hepatocytes were much lower than the control according to the H&E staining (Fig. 6C). Also, the expression of genes involved in TAG synthesis was significantly inhibited, without significant changes in lipolysis (Fig. 6G). Similar to adipose tissue, the inflammatory status induced by HFD was reduced by EPS treatment in livers, as reflected by the mRNA and protein levels of the gene markers of M1-like macrophages and M2-like macrophages (Fig. 6D–F). Overall, the results showed that the accumulation of lipid in the liver was decreased by treatment with EPS with reduced inflammation.

### LGG EPS inhibited adipogenesis in 3T3-L1 cells via Toll-like receptor-2

**Figure 7:**
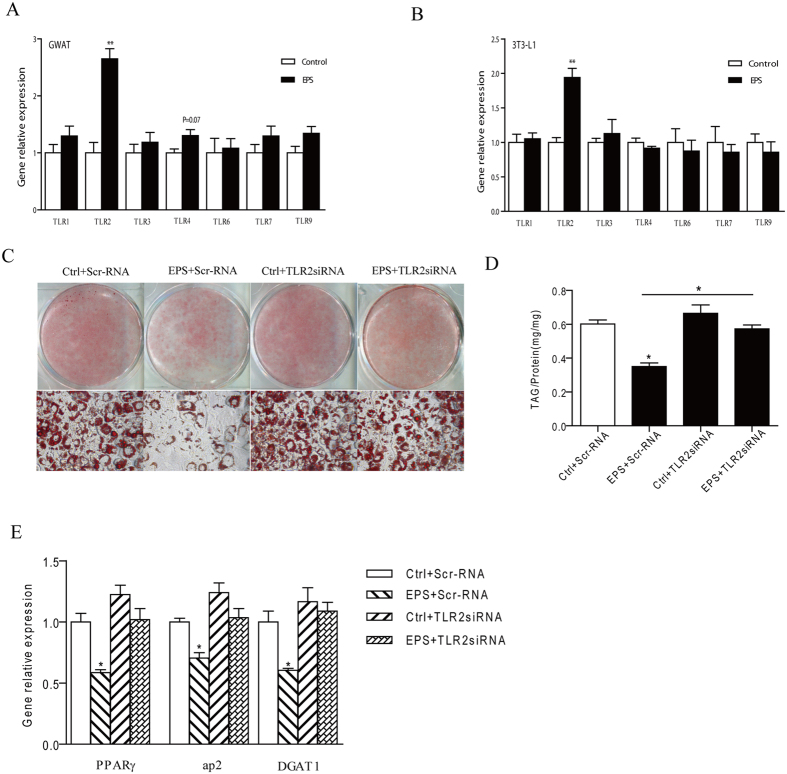
LGG EPS inhibited adipogenesis in 3T3-L1 cells via Toll-like receptor-2. (**A,B**) The effects of LGG EPS on the expression of TLRs in mature 3T3-L1 adipocytes (**A**) and gonadal white adipose tissue of mice (**B**). (**C**) Knockdown of TLR2 expression in 3T3-L1 cells with transfection of siRNA reversed lipid accumulation as assessed by Oil Red O staining. (**D**) Quantification of lipid accumulation of differentiated cells by TAG assay. (**E**) Knockdown of TLR2 reverses the expression of adipocyte markers measured by real-time PCR. Data were expressed as mean ± SEM of three independent experiments (n = 3). Significance was established using a two-tailed Student’s t-test. Differences were considered significant at P < 0.05 (*) and P < 0.01 (**).

Toll-like receptors (TLRs) mediate recognition of microbial patterns, and TLR2 plays an important role in the recognition of bacteria EPS^36^,^37^. To detect the response of TLR family to LGG EPS, the expression of TLR family was determined in mouse adipose tissue and 3T3-L1 cells, in which the inhibited adipogeneisis by EPS was observed (Figs 3 and 5). Interestingly, the expression of TLR2 in adipose tissue from EPS-treated mice was significantly increased, and no changes of other TLR members were observed (Fig. 7A). Consistently, TLR2 expression was also induced in 3T3-L1 cells in response to EPS (Fig. 7B). These results prompted us to investigate the roles of TLR2 in inhibiting adipogenesis by LGG EPS. To show the effects of TLR2, siRNA-mediated TLR2 silencing was performed. Confluent 3T3-L1 cells were transfected with siRNA duplexes 24 h prior to the induction of adipogenesis with MDI. As shown in Supplementary Fig. S7A, nearly 60% expression of TLR2 was knocked down by its siRNA. The inhibited TAG accumulation by EPS was reversed by silencing TLR2 expression (Fig. 7C,D). Furthermore, the expression of adipocyte markers such as PPARγ, ap2, and DGAT1, was also reversed by silencing TLR2 expression (Fig. 7E). These data strongly demonstrated that the effect of EPS on inhibiting lipid accumulation in adipocytes were TLR2 dependent.

TLR2 can function as either a homodimer or heterodimer with TLR1 or TLR6^37^. To show the effects of TLR1 and TLR6, siRNA-mediated TLR1 or TLR6 silencing was performed. Confluent 3T3-L1 cells were transfected with siRNA duplexes 24 h prior to the induction of adipogenesis with MDI. As shown in Supplementary Fig. S7B,C, successful knockdown of the TLR1 and TLR6 can be accomplished using this technique. However, the inhibited TAG accumulation by LGG EPS was not reversed by silencing TLR1 or TLR6 expression (Supplementary Fig. S7D–F). No reverse of TAG accumulation in TLR6- or TLR1-knockdown mature 3T3-L1 adipocytes in the presence of LGG EPS suggests the possible involvement of TLR2 homodimers rather than TLR2-TLR6 or TLR2-TLR1 heterodimers in the regulation of adipocyte adipogenesis.

## Discussion

The present study was focused on the effects of preboiotic components on alleviating lipid metabolic disorder. With the exponential development of gut metagenomics, it is widely accepted that probiotics have the ability to improve host health, such as IBD therapy^38^, improving immunity^39^, diabetes and obesity therapy^40^,^41^,^42^. Those studies were concentrated in the beneficial effects of live probiotic bacteria and their metabolic products. SCFAs, including acetic acids, propionate acids and butyric acids, were reported to exert beneficial effects on hosts as the main bioactive molecules fermented from gut microbiota on fiber^41^,^43^,^44^. Interestingly, the present study focused on EPS, the components of *Lactobacillus rhamnosus* GG, widely known as a probiotics strain. The lipid metabolic discords induced by HFD in systematic lipid homeostasis and peripheral organs, as shown in serum index and lipid metabolism in liver and adipose tissue, was significantly improved by interval EPS intraperitoneal injection. What’s more, the inflammation was also reduced by the EPS injection. It will be a great help to extend the probiotic roles in lipid metabolic disorders through food or medicine intervention, not limited to the prebiotic overseeding which causes gut microbiota imbalance.

The strict evaluation and functional exploration of prebiotic EPS were analyzed based on multiple evaluation models including *in vitro* and *in vivo*, multiple indexes, organs and tissues. The evaluation of LAB cell extracts from 12 strains, based on the cell-model screening, was done to detect the effects on adipogenesis in 3T3-L1 adipocytes like previous reports^30^,^31^. Unfortunately, the inflammation was significantly induced by the LAB cell extracts although the significantly inhibited adipogenesis was observed like other studies. It is indispensible to consider both inflammation status and lipid metabolism when evaluating the beneficial effects. The crude extracts of lactobacilli cell contain a variety of compounds, such as peptidoglycan, lipoproteins, lipopeptides and EPS^36^. Therefore, isolation and purification of EPS from LAB cell extracts was further analyzed based on the existing effects of autoclaved or heated macromolecular fraction from cell extracts. The indexes reflecting inflammation and adipogenesis were detected in adipocytes, adipose tissues, liver and serum. Obviously, the treatment of purified EPS from LGG decreased lipid accumulation with reduced inflammation in the adipocyte and mouse models. Adipose tissue macrophages (ATMs) are classified into classical activated macrophages (also known as M1 macrophages) and alternatively activated macrophages (also known as M2 macrophages). It is well known that M1 macrophages infiltration of WAT triggers inflammation, while M2 macrophages suppress inflammation^45^,^46^. Obviously, purified EPS administration induced the M2 macrophages and inhibited M1 macrophages in adipose tissue and liver, which explained the improved inflammation status by EPS. Therefore, the data could reflect a true status in mice and provide reliable information.

Fungal- and plant-derived polysaccharides displayed encouraging therapeutic potential, and have been proved to inhibit the adipogenesis in 3T3-L1 cells and have the anti-obesity effect *in vivo*^47^,^48^,^49^. In addition, polysaccharides from bacteria could modulate animal immunity^36^,^37^,^50^. Thus, the polysaccharides from different species are potent and more studies are required. No data shows the effects of isolated polysaccharides from lactobacilli or other bacteria on host lipid metabolism to date. We demonstrated that EPS from *Lactobacillus* had the anti-obesity effects, including smaller adipocytes, reduced white fat pad, decreased serum TAG levels and reduced inflammation, compared with its counterpart mice. The increase in adipose tissue which occurs in obesity is due to enlargement of existing fat cells, or to increase in the total number of fat cells, or to a combination of both processes. Adipocyte size is known as an important index as a metabolic disease due to its different adipokine secretion of adipocytes closely related to the adipocyte size^51^. In addition, the distribution of adipose tissue is more significant than the degree of obesity *per se*, as adipose tissue has endocrine functions. It has been shown that abdominal adipose tissue distribution is strongly correlated with the metabolic complications of obesity^51^,^52^. Obviously, LGG EPS injection caused the reduction in adipocyte size and fat pads in abdomal white adipose tissue.

To decipher the molecular mechanism how EPS from LGG modulated lipid metabolisms, we found EPS induced the expression of TLR2 in adipocytes *in vitro* and adipose tissue *in vivo*, and its expression were required to inhibit TAG accumulation by EPS. Polysaccharides A (PSA) from *Bacteroides fragilis* was identified as TLR2 ligands to activate TLR2 signaling, which induced T_reg_ cells to suppress T_H_17 cells and orchestrate immune responses^37^. The present study did not provide direct data to show LGG EPS as the ligands of TLR2, but its TLR2-dependence was obvious. Polysaccharides A (PSA) from *Bacteroides fragilis* was identified as TLR2 ligands to orchestrate immune responses^37^, indicating the reliance of TLR2 mediating EPS in adipocytes in the present study. More details are required to explore the signaling transduction to modulate the adipocyte differention in response to EPS-TLR2 stimuli.

To study the effect of LGG EPS on the lipid metabolism of the host, the LGG EPS solutions (5 mg/ml) were injected i.p. into mice at interval per two days at the dosage of 50 mg/kg for consecutive 14 days. Dose-range and dose-frequency of injections are vital important for polysaccharides to exert its beneficial effect. When the dose was too high or too low, the polysaccharides even showed completely opposite effect^53^. We also found if the LGG EPS solutions were injected i.p. daily into mice at the same dosage for the same processing time, LGG EPS resulted in severe inflammation (data not shown). In addition, the effect of oral administration of EPS is also being studied. We have to note that the mechanisms of EPS injection might be different from oral administration, in which gut microbiota and digesting systems were considered to be involved in.

In summary, our results indicated that the crude extracts of lactobacilli cell walls, because they have complex components were not suitable for evaluating the effect of lactobacilli *in vitro* experiment. The injection of purified ESP (50 mg/kg) from LGG strains systematically improved the lipid metabolisms in HFD-feeding mice, including the decreased TAG levels in serum and liver, decreased fat pads, smaller adipocytes and decreased inflammation. The expression of TLR2 was required for EPS to improve the lipid metabolic disorders. Then, LGG EPS had the potential to develop the drug to intervene lipid metabolic disorders in mammals.

## Materials and Methods

### Bacterial strain and growth condition

All of 12 *Lactobacillus* strains (*L. acidophilus* ATCC4356, *L. rhamnosus* CICC20300, *L. buchneri* CGMCC1.3108, *L. casei* ATCC334, *L. Plantarum* ATCC14917, *L. brevis* ATCC367, *L. johnsonii* ATCC33200, *L. Delbrueckii* ATCC11842, *L. Amylovorus* ATCC 33620, *L. rhamnosus* ATCC53103, *L. casei* BL23, *L. reuteri* ATCC23272), purchased from China Center of Industrial Culture Collection, were stationarily cultivated in de Man, Rogosa and Sharpe broth (MRS broth, containing 10 g/l of tryptone, 10 g/l of beef extract, 5 g/l of yeast extract, 10 g/l of glucose, 5 g/l of sodium acetate·3H_2_O, 1 g/l of tween 80, 2 g/l of citric acid ammonium salt dibasic, 0.2 g/l of MgSO4·7H_2_O, 0.05 g/l of MnSO_4_·H_2_O, and 2 g/l of K_2_HPO_4_) at 37 °C for 48 h. After culturing the LAB, all strains were harvested in a refrigerated centrifuge (12,000 rpm for 10 min at 4 °C) and washed three times with distilled water for the removal of MRS broth.

### Preparation of the cell extract from LAB

The cultured LAB was resuspended in distilled water at a concentration of 10 mg/ml and sonicated 5 seconds with a 10-second interval for 50 times on ice by using a sonicator (Fisher Scientific Co., Toronto, ON, USA) to make the homogeneous cell extracts. The suspension was centrifuged at 12,000 rpm for 15 min at 4 °C. And the resultant supernatants were filter-sterilized (pore size, 0.45 μm), lyophilized, and kept at −80 °C until use. The 3T3-L1 cells were treated with the concentrations of 40 μg/ml of the supernatant^30^.

### The extraction and purification of LGG EPS

Total EPS was extracted from the washed LAB cells by mild sonication (40 W, 10 min) and Water Bath incubation (80 °C, 4 h). The EPS was precipitated by gradually adding cold ethanol to 75% (v/v) to the filtered supernatant for 24 h, followed by centrifugation 12000 rpm for 20 min. The precipitated product was washed and dissolved in water obtained from an Alpha-Q reagent grade water purification system (Millipore Co., Milford, MA). The aqueous solution of the EPS were further treated with sevag reagent at a final concentration of 25% and incubated for 2 h under gentle agitation. Precipitated proteins were removed by centrifugation at 5000 g for 20 min. After centrifugation, the solution containing EPS was dialyzed (molecular weight cut-off: 3000 Da) against 5 liter of distilled water for 2 days with water changes three times per day.

The EPS (8 mg/ml) was further purified by size-exclusion chromatography (SEC) on a column of Superdex75 (10/300 GE) (Pharmacia, Uppsala, Sweden) fitted to an AKTA FPLC system (Pharmacia) and eluted with 0.3 M NaCl buffer. The amount of carbohydrates was estimated by the phenol-sulfuric acid method^54^. The EPS eluting in the void volume was lyophilized.

### Determination of monosaccharide compositions

The monosaccharide composition was determined by TLC and HPAEC. Briefly, 20 mg EPS was hydrolyzed with 2-ml sulfuric acid (1 mol/l) at 100 °C for 4 h. The residual sulfuric acid was removed by neutralization with excessive BaCO_3_ reaction for 12 h. This solution was adjusted to pH 7 and diluted to 20 ml. The hydrolyzate was evaporated under reduced pressure, dissolved in 2-ml ultra pure water. The resulting hydrolyzate was analyzed by TLC and HPAEC.

TLC analysis was conducted according to the previous report^55^. Migration was performed twice on a silica gel TLC plate (20 cm × 20 cm) using n-butanol–methanol–25% ammonia solution–water (5:4:2:1 [vol/vol/vol/vol]). Carbohydrates were visualized by heating the TLC plate after spraying with aniline-diphenylamine reagent (4 ml of aniline, 4 g of diphenylamine, 200 ml of acetone, and 30 ml of 85% phosphoric acid). Galactose, rhamnose and N-acetyl-D-glucosamine were used as standard monosaccharides and baking it at 110 °C for 5 min.

The hydrolysates was injected into a model 2500 HPAEC system (Dionex, Sunnyvale, CA, USA) equipped with a Carbo-Pac PA200 column (3 × 250 mm). The sample in the column was eluted by NaOH (600 mM) at the flow rate of 0.45 mL/min. The monosaccharide compositions were identified by comparing retention times with the standard compounds.

### FT-IR spectroscopy and NMR analysis

The major structural groups of the purified EPS were detected using a Fourier-transformed infrared spectroscopy (Scimitar, America Varian Technology Co. Ltd.). EPS (1–2 mg) was ground with potassium bromide (KBr) powder and pressed into pellets for FT-IR measurement in the frequency range of 4000–400 cm^−1^.

For NMR analysis, the sample (10 mg) was dissolved in 0.5 mL of D_2_O. ^1^H NMR and ^13^C NMR spectra were recorded on a Bruker Spectrometer (600 MHz) at a probe temperature of 30 °C. Prior to analysis, the samples were exchanged twice with D_2_O upon freeze drying. ^1^H and ^13^C NMR chemical shifts are reported with internal D_2_O and methyl alcohol as reference, respectively.

### Molecular weight determination

The EPS molecular weight and its distribution were measured using GPC/MALL^56^. This process was performed on an infusion system (Waters 515 HPLC Unit Pump) fitted with one gel column (Shodex OHpak SB-806M HQ) at 35 °C as column temperature and the combined detectors (Wyatt-DAWN HELEOS® eighteen-angle laser light scattering and Wyatt-Optilab rex refractive index detector). The mobile phase was a 0.1 mol/L NaNO_3_ at a flow rate of 0.5 mL/min. The sample concentration was 0.2 mg/mL and the injection volume was 0.2 mL.

### Cell Culture

3T3-L1 cells (American Type Culture Collection, ATCC) were cultured in DMEM containing high glucose supplemented with 10% fetal bovine serum (FBS) and penicillin/streptomycin in 6-well culture plates. Two days after confluence (day 0), the cells were cultured in the MDI differentiation medium containing 5 mM 3-isobutyl-1-methylxanthine (IBMX), 1 mM dexamethasone (Sigma, USA), and insulin (10 mg/mL) in DMEM supplemented with 10% fetal bovine serum (FBS) for 2 days. On day 2, medium was replaced by mature medium which was DMEM containing 10% FBS and 1 μg/mL insulin and incubated for 2 days, followed by culturing with mature medium for an additional 2 days (day 6), at which time more than 90% of cells were mature adipocytes with accumulated fat droplets. Cells were maintained at 37 °C in a humidified 5% CO_2_ atmosphere.

### Oil red O staining of 3T3-L1 adipocytes

3T3-L1 cells were treated with vehicle or extract or EPS for 6 days during adipogenesis as described above. Thereafter 3T3-L1 cells were fixed with 4.0% formaldehyde (Sigma) in PBS(−) and stained with Oil red O. After cells were mounted with glycerol gelatin, images for each dish were captured using a Scanner and a microscope (Leica DMIL-LED, Germany).

### TAG assay

For cellular TAG level determination, lipids were extracted from 6-day differentiated 3T3-L1 adipocytes as previously described^57^, dissolved in isopropanol and determined by serum TAG determination kit (Sigma). The protein concentration was determined by using a Bradford reagent (Sigma, St. Louis, MO). TAG contents were normalized to the protein amounts of each sample.

Tissues were homogenized in PBS buffer with protease inhibitors. A chloroform/methanol (2:1) solution was rapidly added to the homogenate and the samples were vortexed. The samples were centrifuged at 250 g for 10 min, to separate the phases. The lower lipid-containing phase was carefully aspirated and allowed to dry in a 70 °C metal bath with nitrogen steam. The dried lipids were emulsified in chloroform with 5% Triton X-100. Finally, dried emulsified lipids with nitrogen gas were reconstituted in distilled water. The contents of TAG and cholesterol ester were measured by enzymatic reaction according to the instruction manual (Wako Diagnotics, Japan).

### Animals, Diets and Experimental Design

The animal experiments were carried out in the animal centre of Tsinghua University. Procedures involving animals were performed in accordance with Chinese legislation associated with animal experimentation and the studies were approved by the Institutional Animal Care and Use Committee of Tsinghua University (12-LP-1). C57BL/6J Male mice with six-week-old were fed with a normal chow diet (ND, 5053, PicoLab Rodent Diet 20) for the experimental treatments with free access to water. The initial average body weight was similar among 4 different treatments (n = 6). 50 mg/kg body weight of EPS (5 mg/ml) in saline or the similar volume saline as control was injected into the mouse intraperitoneal once every two days for total 2 weeks^50^,^53^ fed NDs or HFDs (D12331, Research Diet, USA). Body weight and food consumption was recorded every two days. After the 2-week feeding and injection period, mice were fasted for 12 hours and euthanized for sample collection. The weight of visceral organs (liver, spleen, and kidney) and subcutaneous, perirenal, and epididymal white adipose tissues were measured. Mouse experiments were performed in the animal facility of the Center of Biomedical Analysis at Tsinghua University (Beijing, China). The laboratory animal facility has been accredited by the Association for Assessment and Accreditation of Laboratory Animal Care International.

### Histopathology of liver and gonadal white adipose tissue (GWAT)

Mouse liver and GWAT were rinsed with sterilized phosphate buffer saline (PBS), fixed in 10% formalin in PBS, and then embedded in paraffin for staining with hematoxylin and eosin (H&E). Images were obtained under a microscope (Leica DMIL-LED, Germany). For immunohistochemistry, formalin-fixed and paraffin-embedded sections were blocked with endogenous peroxidase (3% H_2_O_2_ in 80% methanol) for 20 min. Antigen retrieval was performed in 10 mM sodium citrate in a microwave for 15 min. After blocking nonspecific antigen with normal goat serum for 30 min, the slides were then incubated with arginase 1 (Bioworld, BS5618, 1:200 dilution), CD11c (Bioworld, MB8575, 1:200 dilution), or iNOS (Bioworld, BS1186, 1:200 dilution) antibody overnight at 4 °C. The slides were then incubated with biotinylated-labelled secondary antibodies (1:200, GE Health, UK) for 30 min at room temperature. Visualization was performed using 0.1% 3,3′-diaminobenzidine (Dako, Denmark) in PBS together with 0.05% H_2_O_2_^58^.

### Quantitative PCR analysis

Total RNA was isolated from 3T3-L1, mouse livers and GWAT with TRIzol (Invitrogen) extraction. First-strand complementary DNA synthesis was performed using the Superscript First-Strand Synthesis System (Invitrogen). Quantitative real-time PCR reaction were performed using the Power SYBR Green PCR Master Mix (Applied Biosystems) on an ABI 7500 (Applied Biosystems) with reaction volumes of 20 μl. The primer sequences are listed in Supplementary Table S1.

### Transfection of small interfering RNA

3T3-L1 preadipocytes were transfected with siRNA oligonucleotide duplexes 1 day postconfluence with Lipofectamine 3000 (Invitrogen). Generally, 100 pM siRNA was transfected with 5 μl of Lipofectamine/well of a 6-well plate with fresh medium. *TLR2-, TLR6-, TLR1*-specific siRNAs and scramble controls were synthesized by GenePharma (Shanghai, China). The sense sequence of *TLR2-, TLR6-, TLR1*-specific siRNA were 5′-CCGCUCCAGGUCUUUCACCUCUAUU-3′, 5′-CCAAUACCAC-CGUUCUCCAUUUGGU-3′, and 5′-GACAUCCUCUCAUUGUCCAAGCUGA-3′, respectively^59^. The sense sequence of control non-specific TLR2, TLR6, TLR1 scramble RNA were 5′-ACCUCGUUCUCUCCGUAUUGCCUAC-3′, 5′-GACUGCCCCUUUACUCCAUUGACUA-3′, and 5′-GUACCACUCGAUACG-CCGUCUUAAU-3′, respectively. Cells were treated with siRNA for 24 h, and then cells were processed using differentiation protocols as described above.

### Statistical analysis

The statistical data reported includes results from at least three biological replicates. All results are expressed as mean ± SEM. All statistical analyses were performed in GraphPad Prism Version 5 (GraphPad Software). Significance was predominantly established using a two-tailed Student’s t-test. Differences were considered significant at P < 0.05 (*) and P < 0.01 (**).

## Additional Information

**How to cite this article**: Zhang, Z. *et al*. Isolated exopolysaccharides from *Lactobacillus rhamnosus* GG alleviated adipogenesis mediated by TLR2 in mice. *Sci. Rep.* **6**, 36083; doi: 10.1038/srep36083 (2016).

**Publisher’s note:** Springer Nature remains neutral with regard to jurisdictional claims in published maps and institutional affiliations.

## Supplementary Material

Supplementary Information
