# Supplementary material for: Isolated exopolysaccharides from Lactobacillus rhamnosus GG alleviated adipogenesis mediated by TLR2 in mice
Source: Sci Rep. 2016 Oct 27;6:36083. doi: 10.1038/srep36083 (PMC5081535; doi:10.1038/srep36083)
Supplement: Supplementary Information [file srep36083-s1.doc]

**Supplementary information**

Isolated exopolysaccharides from *Lactobacillus rhamnosus* GG alleviated adipogenesis mediated by TLR2 in mice

Zhen Zhang^a^, Zhigang Zhou^a*^,Yu Li^a^, Linkang Zhou^b^, Qianwen Ding^a^, Li Xu^a,b*^

^a^ Key Laboratory for Feed Biotechnology of the Ministry of Agriculture, Feed Research Institute, Chinese Academy of Agricultural Sciences, Beijing, P. R. China.

^b^ MOE Key Laboratory of Bioinformatics and Tsinghua-Peking Center for Life Sciences, School of Life Sciences, Tsinghua University, Beijing 100084, P. R. China.

* Corresponding authors

No. 12 Zhongguancun South Street, Beijing 100081, PR China. Tel.: +86 10 82106073; Email: zhouzhigang03@caas.cn (Zhou Z); xulilulu@tsinghua.edu.cn (Xu L)

**Supplementary methods**

**Isolation of stromal vascular fraction (SVF) cells from subcutaneous and gonadal fat depots**

SVF cells were harvested as previously reported [1, 2]. Subcutaneous and gonadal adipose tissue of C57BL/6J mice was incubated in Hank’s balanced salt solution (HBSS; Sigma-Aldrich) containing 0.2% collagenase type 2 (Worthington) for 45 minutes at 37°C with constant shaking. After inactivating collagenase activity with 10% fetal bovine serum (FBS) in Dulbecco’s modified eagle medium (DMEM), the cell suspension was filtered through a 40 μm nylon mesh (BD Biosciences), followed by centrifugation at 420 g for 5 minutes. Floating adipocytes and supernatant were removed from the SVF pellet. The SVF pellet was washed and resuspended in the medium. Extra precaution was taken during repeated washing with medium considering the nature of adipocytes in developing subcutaneous and gonadal adipose tissue, which have tiny lipids that are not large enough to be separated by buoyancy, and are thus susceptible to contamination.

SVF cells were seeded on a 6-well plate and cultured in DMEM containing 10% FBS (culture medium). Two days after confluence (day 0), culture medium was replaced with induction medium [5 mg/ml insulin, 1 mM dexamethasone, 0.5 mM 3-isobutyl-1-methylxanthine (IBMX)] to induce adipogenesis. After 2 days (day 2), induction medium was replaced with maintenance medium (5 mg/ml insulin was added to the culture medium) and changed every 2 days until analysis (day 6). To confirm adipocyte differentiation, Oil-Red O was used.

[1] Han, J. et al. Adipose tissue is an extramedullary reservoir for functional hematopoietic stem and progenitor cells. *Blood* **115**, 957-964 (2010).

[2] Han, J.et al The spatiotemporal development of adipose tissue.*Development* **138**(22), 5027-5037 (2011).

**Cell proliferation analysis**

3T3-L1 cell proliferation was determined by counting the cells with a hemocytometer. 3T3-L1 cells were seeded at a density of 5 × 10^4^ cells/ml and cultured in 6-well plated in DMEM culture medium until confluent. 2 days after confluent, they were incubated in a differentiation medium supplemented with (or without) 10.0 μg/ml LGG EPS. At indicated time points, cells were harvested and counted using a hemocytometer. Under microscope examination, cells were counted from four different fields of the hemocytometer. Dead cells were excluded by Trypan Blue staining.

Supplementary Table S1: Sequences of primers used for qRT-PCR analysis.

| Gene Name | Forward (5’-3’) | Reverse(5’-3’) |
| --- | --- | --- |
| β-actin | CATCCGTAAAGACCTCTATGCCAAC | ATGGAGCCACCGATCCACA |
| 28S | CGCCATCATGGACACGAGTC | AAGATTAACGCAACCTTCGAG |
| FAS | ATCCGGAACGAGAACACGATCT | AGAGACGTGTCACTCCTGGACTT |
| PPARγ | AACTCTGGGAGATTCTCCTGTTGA | GAAGTGCTCATAGGCAGTGCAT |
| C/EBPα | AGGTGCTGGAGTTGACCAGT | CAGCCTAGAGATCCAGCGAC |
| aP2 | CATGGCCAAGCCCAACAT | CGCCCAGTTTGAAGGAAATC |
| HSL | GCTGGGCTGTCAAGCACTGT | GTAACTGGGTAGGCTGCCAT |
| ATGL | CATCTCCCTGACTCGTGTTTC | CAAGTTGTCTGAAATGCCGC |
| TNFα | GCCACCACGCTCTTCTGCCT | GGCTGATGGTGTGGGTGAGG |
| MCP 1 | TCTGGACCCATTCCTTCTTG | AGGTCCCTGTCATGCTTCTG |
| IL-6 | CCAGAGATACAAAGAAATGATGG | ACTCCAGAAGACCAGAGGAAAT |
| Arginase 1 | ATGGAAGAGACCTTCAGCTAC | GCTGTCTTCCCAAGAGTTGGG |
| MGL | ATGATGTCTGCCAGAGAACC | ATCACAGATTTCAGCAACCTTA |
| Clec7a | AGGTTTTTCTCAGTCTTGCCTTC | GGGAGCAGTGTCTCTTACTTCC |
| SCD1 | CCCTCCGGAAATGAACGAGAG | GCCGGGCTTGTAGTACCTC |
| LPL | TGGAGAAGCCATCCGTGTG | TCATGCGAGCACTTCACCAG |
| DGAT1 | ACCGCGAGTTCTACA | AGGGGAACGCTCACTAGGTA |
| TLR1 | TGAGGGTCCTGATAATGTCCTAC | AGAGGTCCAAATGCTTGAGGC |
| TLR2 | GCAAACGCTGTTCTGCTCAG | AGGCGTCTCCCTCTATTGTATT |
| TLR3 | AGCTTTGCTGGGAACTTTCA | GAAAGATCGAGCTGGGTGAG |
| TLR4 | TTCAAGACCAAGCCTTTCAG | CATAGTCCT TCCATGATAGA |
| TLR5 | GCAGGATCATGGCATGTCAAC | ATCTGGGTGAGGTTACAGCCT |
| TLR6 | ACACAATCGGTTGCAAAACA | GGAAAGTCAGCTTCGTCAGG |
| TLR7 | CCACAGGCTCACCCATACTTC | GGGATGTCCTAGGTGGTGACA |
| TLR8 | GTTTCCTCGTCTCGAGTTGC | TCAAAGGGGTTTCCGTGTAG |
| TLR9 | ATGGTTCTCCGTCGAAGGACT | GAGGCTTCAGCTCACAGGG |

Supplementary Table S2 Molecular weight and relative molecular weight distribution of LGG EPS (mean ± RSD)

| Molar mass moments (g/mol) | | | | Polydispersity | |
| --- | --- | --- | --- | --- | --- |
| Mn^a^ | Mp^b^ | Mw^c^ | Mz^d^ | Mw/Mn | Mz/Mn |
| 180,900± 0.01 | 188,600± 0.01 | 229,300± 0.008 | 331,800± 0.01 | 1.268 ± 0.01 | 1.835± 0.02 |

^a^ Number-average molecular weight.
^b^ Peak-position molecular weight.
^c^ Weight-average molecular weight.
^d^ Z-average molecular weight.


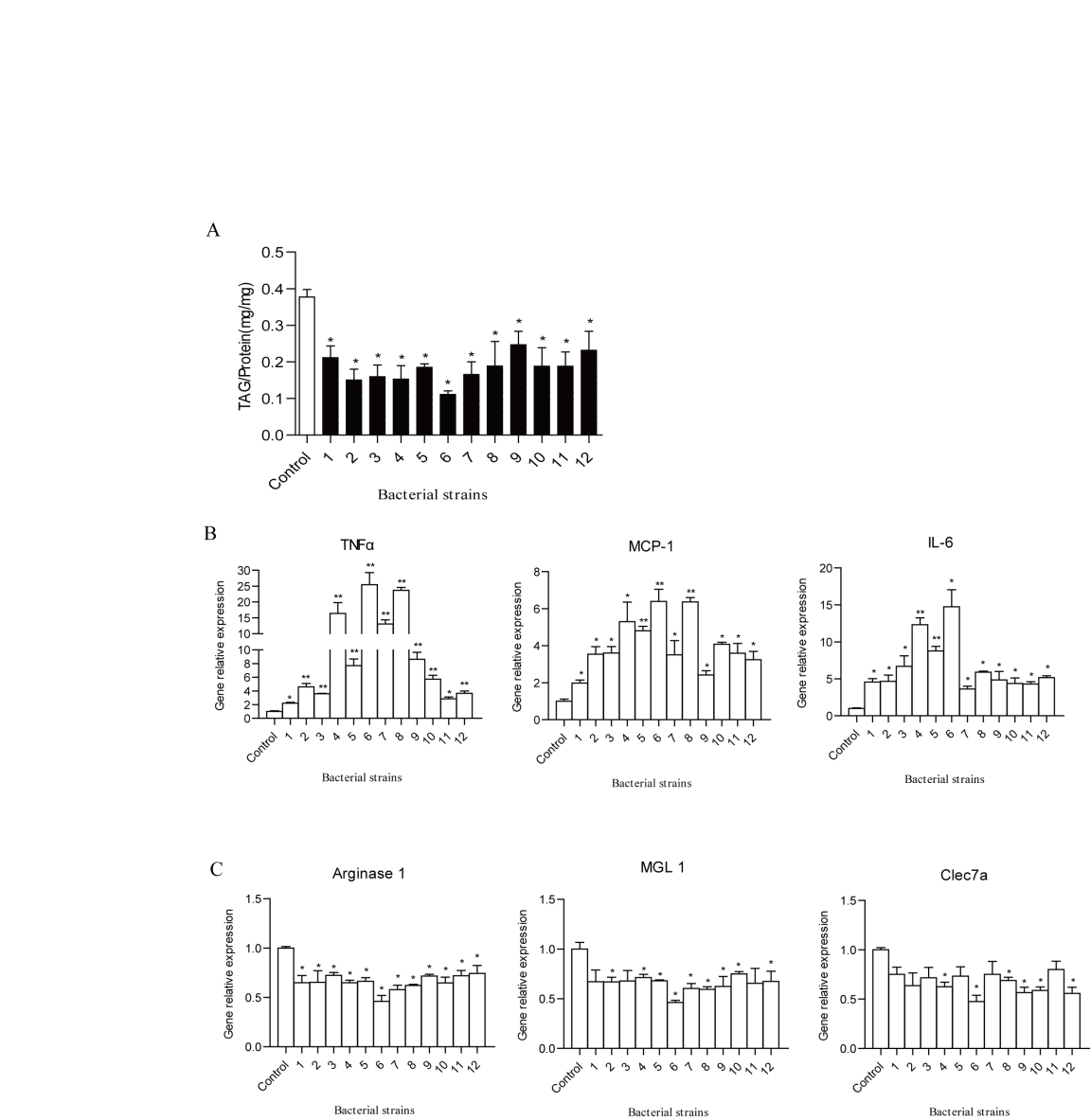


**Supplementary Figure S1.** **The effects of cell extract from 12 *Lactobacillus* strains on the adipogenesis** **(A) and inflammation (B&C) in 3T3-L1 adipocytes.** 3T3-L1 cells from initiating differentiation (Day 0) to terminate mature (Day 6) as indicated in methods were treated with the supernatants of cell extracts (40 μg/ml) from 12 Lactobacillus strains or sterile deionized water as the control. The LAB strains from 1 to 12 were respectively *L. rhamnosus* ATCC53103 (LGG), *L. acidophilus* ATCC4356, *L. rhamnosus* CICC20300, *L. buchneri* CGMCC1.3108, *L. casei* ATCC334, *L. Plantarum* ATCC14917, *L. brevis* ATCC367, *L. johnsonii* ATCC33200, *L. Delbrueckii* ATCC11842, *L. Amylovorus* ATCC 33620, *L. casei* BL23, *L. reuteri* ATCC23272. (A) Effects of cell extracts from 12 candidate Lactobacillus strains on TAG accumulation in 3T3-L1 adipocyte. (B&C) The expression of M1 proinflammatory genes (B) and M2 anti-inflammatory genes (C) in 3T3-LI cells by q-PCR. Data were expressed as mean ± SEM of three independent experiments (n=3). Significance was established using a two-tailed Student’s t-test. Differences were considered significant at *P* < 0.05 (*) and *P* < 0.01 (**).


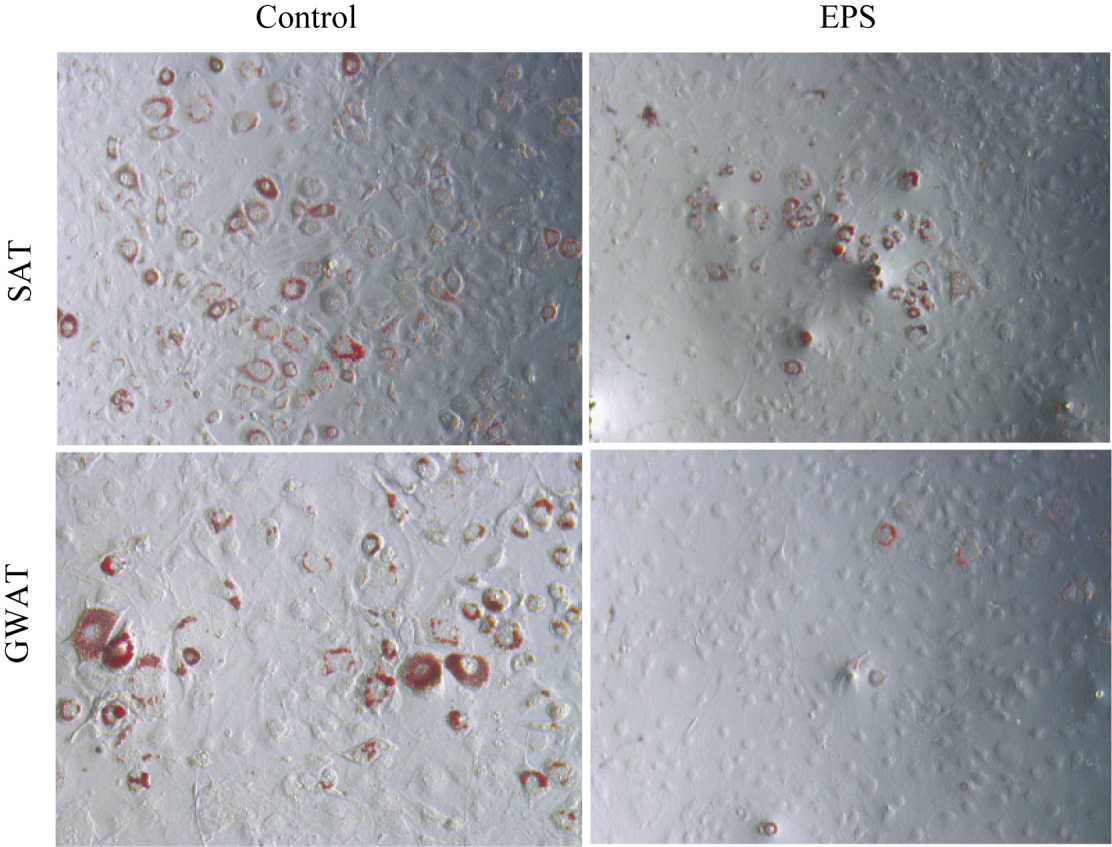


**Supplementary Figure S2. Inhibited adipogenesis in primary preadipocytes from subcutaneous and visceral fat depots by LGG EPS.**

The primary preadipocytes from subcutaneous (SAT) and gonadal (GWAT) fat depots was treated by isolated EPS (10 μg/ml) from LGG during the Day 0-6 and the sampling was done on Day 6 for Oil red O staining.


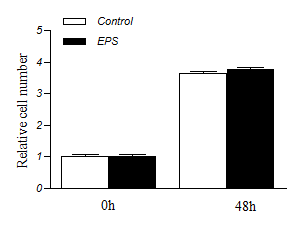


**Supplementary Figure S3. Clonal expansion of 3T3-L1 preadipocytes were not affected by the presence of LGG EPS.** 3T3-L1 preadipocytes were induced to differentiation as normal culture condition in the presence or absence of EPS (10 μg/ml). Cell numbers were counted at the indicated time points after MDI-induced differentiation initiation (0 h and 48 h). The cells were trypsinized and aliquots were taken in triplicate and the numbers were counted by a hemocytometer. Data were shown as change folds referred to the cell number at 0 h time point of control group set as 1. All values were presented as mean ± SEM. Differences were considered significant at *P* < 0.05 (*) and *P* < 0.01 (**).

A


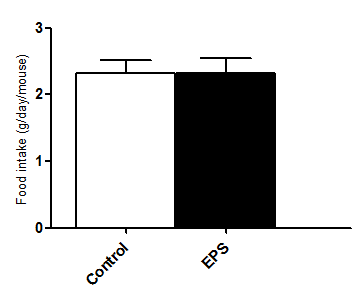

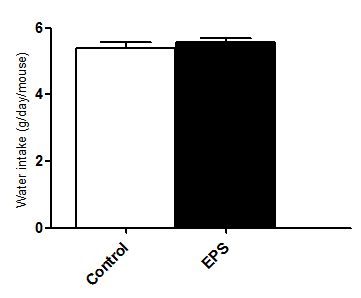


B

**Supplementary Figure S4. The food (A) and water (B) intake of HFD-feeding C57BL/6J mice with saline or EPS injection.**

A

B

C


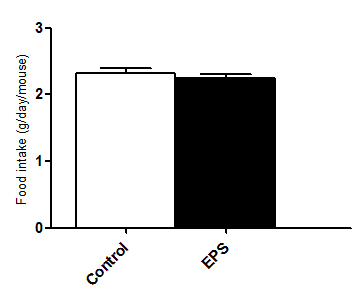

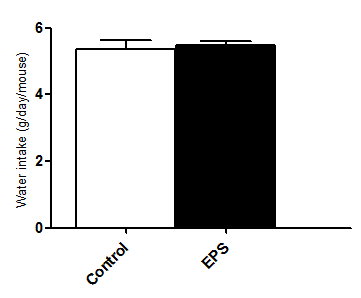


D

**Supplementary Figure S5. The effects of EPS injection in ND-feeding C57BL/6J mice.**

There were no significant differences observed in body weight (A), food intake (B) and water intake (C) monitored every 2 days after EPS injection. (D) Effects of EPS injection on organ coefficients in ND-feeding C57BL/6J mice (n = 6).


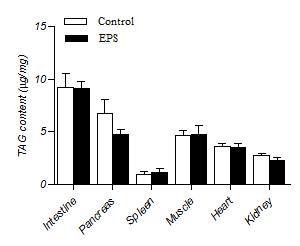


**Supplementary Figure S6. The relative contents of TAG in muscles, intestines, pancreases, spleens, hearts, and kidneys.**


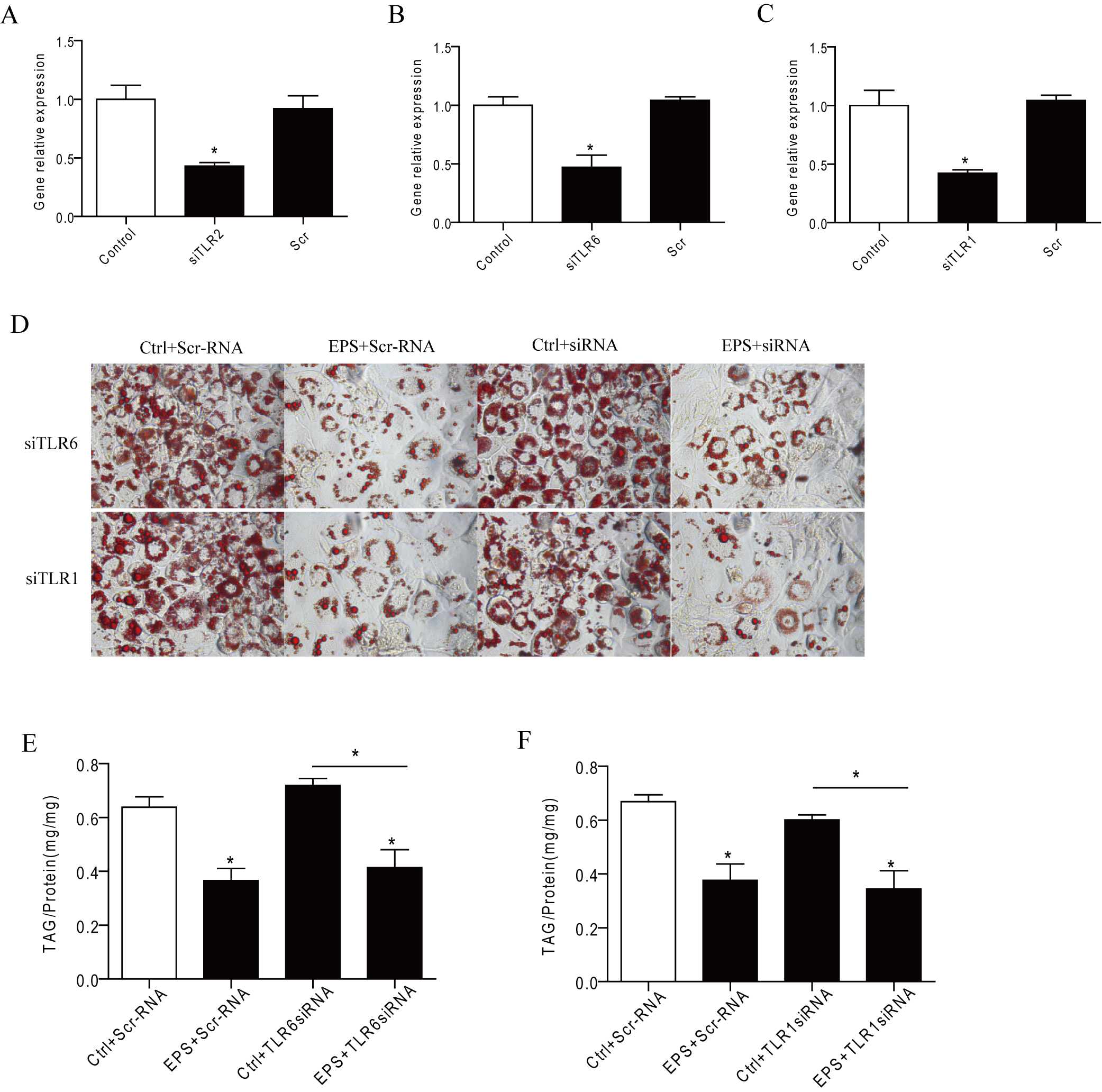


**Supplementary Figure S7. Knock-down of TLR2，TLR6 or TLR1 levels in 3T3-L1 cells using siRNA.** 1-day postconfluent 3T3-L1 cells were transfected with (A) TLR2 siRNA duplex, (B) TLR6 siRNA duplex or (C) TLR1 siRNA duplex. Twenty-four hours later (2 days postconfluence), differentiation mixture was added, and RNA were isolated 48 h later. (D) The profile of lipid droplet formation with Oil red O staining in TLR6- or TLR1-knockdown mature 3T3-L1 adipocytes. TAG accumulation in (E) TLR6- or (F) TLR1-knockdown mature 3T3-L1 adipocyte by TAG assay. Data were expressed as mean ± SEM of three independent experiments (n=3). Significance was established using a two-tailed Student’s t-test. Differences were considered significant at *P* < 0.05 (*) and *P* < 0.01 (**).
